# Role of physical activity and fitness on sleep in sedentary middle-aged adults: the FIT-AGEING study

**DOI:** 10.1038/s41598-020-79355-2

**Published:** 2021-01-12

**Authors:** Sol Mochón-Benguigui, Almudena Carneiro-Barrera, Manuel J. Castillo, Francisco J. Amaro-Gahete

**Affiliations:** 1 EFFECTS-262 Research Group, Department of Medical Physiology, School of Medicine, University of Granada, 18016 Granada, Spain; 2 Sleep and Health Promotion Laboratory, Mind, Brain and Behaviour Research Centre (CIMCYC), University of Granada, 18011 Granada, Spain

**Keywords:** Sleep disorders, Disease prevention

## Abstract

The association of physical activity and fitness with sleep still remains unclear since there is a lack of studies in this field of research using objective measurements of these variables. This study aimed to investigate the association of objectively-measured sedentariness, physical activity levels, and physical fitness with sleep quantity and quality in sedentary middle-aged adults. A total of 74 volunteers (52.7% women; aged 53.7 ± 5.1) were recruited for the present study. Cardiorespiratory fitness was measured through a maximal treadmill test, and muscular strength by extension and flexion peak torque, and by the hand grip test. Physical activity and objective sleep parameters were determined through accelerometry, and subjective sleep by the Pittsburgh Sleep Quality Index (PSQI). Reduced levels of sedentariness, greater *V*O_2max_, and greater muscular strength were positively related to improved objective sleep quantity and quality (all *P* ≤ 0.05). Furthermore, higher levels of overall physical activity, *V*O_2max_, and muscular strength were related to better subjective sleep quantity and quality (all *P* ≤ 0.05). Reduced sedentariness and increased physical activity and fitness may be a potential prevention and/or treatment pathway to reduce sleep disturbances and, in general, to improve patients physical and psychological health for a successful aging process.

## Introduction

Regular physical activity has been widely shown to be a well-established protective factor related to the prevention and management of a vast number of severe pathological conditions^1^ such as obesity^2^, type II diabetes mellitus^3^, life-threating cardiovascular diseases^4^, degenerative neurological disorders^5^, and other adverse chronic illnesses^6,7^. Programmed physical activity (i.e. exercise) is an effective strategy to fight against several cardiometabolic diseases through physical fitness improvements, since its main components (i.e. cardiorespiratory fitness and muscular strength) are considered powerful predictors of physical and psychological health and all-cause mortality^8–10^.

Similarly, sleep is a widely accepted key component of physiological restitution^11,12^, essential for mental and physical health, and thus general well-being^13,14^. According to epidemiological studies, the prevalence of sleep disorders in the overall population has dramatically increased in the last decade^15^, becoming an economic and clinical burden on the health system, with costs of up to $7494 million per year (Australia, 2004)^16^. Insomnia and obstructive sleep apnoea, the most common sleep disorders with a prevalence of 10–40% and 9–38% in the overall population^17,18^, respectively, have been shown to be related to the development and worsening of a wide-range of medical conditions such as obesity^19^, type II diabetes mellitus^20^, other cardio-metabolic alterations^21,22^, chronic kidney disease^23^, and anxiety and mood disturbances/depression^24^, among others.

Research in this field has shown that increased physical activity may be highly effective at improving sleep quantity and quality, and thus an alternative treatment for sleep disorders/disturbances^25–29^. However, at the current time, the evidence is still limited as to whether physical activity, physical fitness, or sedentary behaviour are more strongly associated with sleep quantity and quality. Furthermore, the majority of these studies have assessed physical activity levels using subjective measures (i.e. self-reported questionnaires)^30^, instead of objective methods such as accelerometry, which is currently considered the gold-standard^31^. Likewise, although previous studies have reported a positive relationship of cardiorespiratory fitness and muscular strength with sleep quantity and quality^32–35^, physical fitness has usually been measured by indirect field test assuming therefore potential bias. Furthermore, although sedentary behaviour, poor physical activity levels, and a decline of physical fitness seem to be indicators for sleep status, to the best of our knowledge, there are no studies investigating the association of sedentariness, physical activity, and physical fitness measured by gold standard methods with sleep quantity and quality in sedentary middle-aged adults.

Therefore, the aim of the present study was to investigate the association of sedentariness, physical activity levels, and physical fitness with sleep quantity and quality in sedentary middle-aged adults. We hypothesized that low levels of sedentariness, and higher levels of both physical activity and fitness would be positively associated with better sleep quantity and quality.

## Materials and methods

### Study protocol and participants

The study population consisted of 74 sedentary middle-aged adults (52.7% women) aged 40–65 years. Participants were enrolled in the FIT-AGEING study^36^, an exercise-based randomised controlled trial (clinicaltrial.gov: ID: NCT03334357, registration date: 07/11/2017), approved by the Human Research Ethics Committee of the “Junta de Andalucía” [0838-N-2017]. An extended explanation of the study methodology can be found elsewhere^36^. All participants signed a written informed consent form, and underwent a complete medical and physical examination prior to participation. Inclusion criteria were: (1) to present a body mass index (BMI) between 18.5 and 35 kg/m^2^, (2) to have a stable weight over the previous 3 months, and (3) to be sedentary (i.e. < 20 min of vigorous-intensity physical activity < 3 days/week). Participants with a diagnosis of any physical or psychological disease and/or medical treatment, as well as pregnancy, were excluded from the study. The study complied with the ethical principles described in the Declaration of Helsinki.

### Measurements

#### Anthropometry and body composition

Body weight and height were measured using a seca model 799 electronic scale and stadiometer (seca, Hamburg, Germany). BMI was calculated as ${\text{Weight }}\left( {{\text{kg}}} \right){\text{/Height}}^{{2}} { }\left( {{\text{m}}^{{2}} } \right)$.

A dual-energy X-ray absorptiometry scanner (Hologic, Inc., Bedford, MA, USA) was used to determine fat mass and lean mass. Fat mass index (FMI) and lean mass index (LMI) were calculated as: ${\text{Fat mass}} \left( {{\text{kg}}} \right)/{\text{Height}}^{{2}} \left( {{\text{m}}^{{2}} } \right),$ and ${\text{Lean mass}} \left( {{\text{kg}}} \right) /{\text{Height}}^{2} \left( {{\text{m}}^{{2}} } \right)$, respectively.

#### Physical activity and sedentary time

Physical activity and sedentary time were determined using a wrist-worn accelerometer (ActiGraph GT3X +, Pensacola, FL, USA) continuously 24 h a day for 7 consecutive days^36^. Participants were instructed to wear the accelerometer on the non-dominant wrist, and to remove it when swimming or bathing. They were provided with a 7-day sleep diary to register bedtime, wake up time, and the time they removed the device each day. The accelerometer was initialised to store raw accelerations at a sampling frequency of 100 Hz^31^. ActiLife software (version 6.13.3, ActiGraph, Pensacola, FL, USA) was used to download the stored data. GT3X + files were subsequently converted to 1 s epoch csv files containing x, y and z vectors to facilitate raw data processing. GGIR package (version 1.5-12, https://cran.r-project.org/web/packages/GGIR/) was selected to process these files in R (version 3.1.2, https://www.cran.r-project.org/). Signal processing included auto-calibration using local gravity as a reference, detection of sustained abnormal high accelerations, detection of non-wear time, calculation of the Euclidean Norm Minus One (ENMO), calculation of waking and sleeping time by an automatized algorithm, determination of sedentary time, light physical activity (LPA) time, moderate physical activity (MPA) time, vigorous physical activity (VPA) time, and moderate-vigorous physical activity (MVPA) time using age-specific thresholds for ENMO, and determination of abnormal high values and detected non-wear time. Only the participants wearing the accelerometers for ≥ 16 h/day during at least 4 of 7 possible days (including at least 1 weekend day) were included in the analysis.

#### Cardiorespiratory fitness

Cardiorespiratory fitness was assessed by maximal oxygen uptake (*V*O_2max_) through a maximal treadmill (h/p/cosmos pulsar treadmill, h/p/cosmos sport and medical gmbh, Germany) test using the modified Balke protocol. After a standardized warm-up (i.e. 1 min at 3 km/h and 2 min at 4 km/h) the treadmill was set at a constant speed (5.3 km/h), increasing 1% per minute until volitional exhaustion. The criteria for considering *V*O_2max_ were: (1) a respiratory exchange ratio ≥ 1.1, (2) a plateau in oxygen uptake (VO_2_) (change of < 100 ml/min in the last 30 s stage), (3) a heart rate within 10 beats/min of the age-predicted maximal heart rate using an age-based equation (i.e. 209–0.73 × age), and (4) a rating perceived exertion peak greater than 18 in the 6–20 Borg scale. The peak oxygen uptake value was considered when these criteria were not fulfilled. VO_2_ and carbon dioxide output (VCO_2_) were assessed using a breath by breath gas analyzer (CPX Ultima CardiO2, Medical Graphics Corp., St Paul, USA) equipped with an oronasal mask (model 7400, Hans Rudolph Inc., Kansas City, MO, USA) and a prevent metabolic flow sensor (Medgraphics Corp., Minnesota, USA). The gas analyser was calibrated before each maximal test. Heart rate was registered every 5 s with a heart rate monitor watch (Polar RS300, Kempele, Finland). Participants were instructed to refrain from caffeine for the previous 24 h, to fast for 3 h before the test, and to avoid any moderate and/or vigorous physical activity during the 24 or 48 h, respectively, prior to the assessment day.

#### Muscular strength

Knee flexion and extension peak torque were assessed applying an isokinetic strength test using a Gymnex Iso-2 dynamometer (Easytech S.r.l., Italy). Participants were seated for testing in the dynamometer’s chair with the backrest angle at 90°, and limbs and hips stabilized with safety belts. The lateral epicondyle of their knee was aligned with the axis of the dynamometer’s resistance lever, and the force pad was placed 3–4 cm above to the medial malleolus. The maximum extension angle was fixed at 170° to avoid knee hyperextension, and the maximum flexion angle was established at 90°. Each participant performed 5 submaximal knee flexions and extensions followed by 3 maximal repetitions, with a resting interval of 60 s between submaximal and maximal trials following a previously validated protocol. The flexion and extension peak torque were determined as the single repetition with the highest muscular force output (Nm). Test–retest reliability, calculated using the intra-class correlation coefficient, resulted higher than 0.90.

Handgrip strength was measured using a digital hand dynamometer (T.K.K. 5401 Grip-D; Takei Scientific Instruments Co., Ltd, Tokyo, Japan) with the scores recorded to the nearest 0.1 kg. Participants were asked to perform the test in a standing position with the forearm slightly separated from their torso at the level of the thigh, and to apply the maximum grip strength gradually and continuously for at least 2 s. Two repetitions were registered with both right and left hands alternatively, with a resting interval of 60 s between trials. The grip span of the dynamometer was fixed at 5.5 cm for men, and an individual hand size adjustment in women was used following a previous study^37^.

Muscular strength and *V*O_2max_ assessments were determined on a different day (separated by 3–7 days) applying similar preconditions.

### Sleep quantity and quality

Objective sleep quantity and quality was determined by accelerometry (see specific details about the procedure above). Wake after sleep onset (WASO; the sum of wake times from sleep onset to the final awakening), total sleep time (TST; total amount of time spent in bed minus sleep onset latency), and sleep efficiency (SE; percentage of sleep time over the bedtime), were calculated from actigraphy recordings. Participants who registered ≥ 16 h/day of wear time for at least 4 out of 7 days (including 1 weekend day) were included in the final analysis.

Subjective sleep quantity and quality was evaluated by the Spanish version of the Pittsburgh sleep quality index (PSQI) scale^38^. This scale consists of 19 self-rated questions grouped into 7 components, each equally scored on a 0–3 scale: (1) subjective sleep quality, (2) sleep latency, (3) sleep duration, (4) habitual sleep efficiency, (5) sleep disturbances, (6) use of sleeping medication, and (7) daytime dysfunction^38^. Global PSQI score is obtained by the sum of the 7 components (ranged from 0 to 21). Lower score indicates better sleep quality whereas scores higher than 5 are associated with poor sleep quality.

### Statistical analysis

Shapiro–Wilk test, visual check of histograms, Q-Q, and box plots were used to check the variable distribution. Descriptive parameters were presented as mean and standard deviation. Given that no sex interactions were observed, the results for men and women were analysed together.

Simple linear regression models were performed to study the association of sedentariness and physical activity levels (sedentary time, LPA, MPA, VPA, MVPA, overall physical activity) and physical fitness (*V*O_2max_, flexion peak torque, extension peak torque and total handgrip strength) with sleep quantity and quality (global PSQI score, TST, WASO, and SE).

Multiple linear regression models were performed to test these associations after adjusting by age, fat mass percentage, FMI, LMI and alcohol intake.

All analyses were conducted using the Statistical Package for Social Sciences (SPSS, v. 23.0, IBM SPSS Statistics, IBM Corp., Armonk, NY, USA) and the level of significance was set at ≤ 0.05. All graphical presentations were created using GraphPad Prism 6 (GraphPad Software Inc., San Diego, CA, USA).

## Results

Participant’s characteristics are shown in Table 1. Poor subjective sleep quality (PSQI > 5) was identified in 40.3% of our cohort.

**Table 1:** Descriptive characteristics of participants.

	N	Mean	SD
Age (years)	74	53.7	(5.1)
**Antropometry and body composition**			
Height (cm)	74	167.8	(9.8)
Weight (kg)	74	75.7	(15.0)
Body mass index (kg/m^2^)	74	26.7	(3.8)
Fat mass (%)	74	39.9	(9.1)
Fat mass index (kg/m^2^)	74	10.7	(3.1)
Lean mass index (kg/m^2^)	74	15.2	(2.9)
**Sleep quantity and quality**			
Total sleep time (min)	71	359.9	(48.8)
Wake after sleep onset (min)	71	63.9	(27.4)
Sleep efficiency (%)	71	85.0	(6.3)
Global PSQI score	67	5.6	(3.5)
**Physical activity**			
Sedentary time (min/day)	71	745.9	(84.2)
Light physical activity time (min/day)	71	173.9	(45.1)
Moderate physical activity time (min/day)	71	94.4	(34.8)
Vigorous physical activity time (min/day)	71	1.7	(2.2)
Moderate-vigorous physical activity time (min/day)	71	96.1	(35.4)
Overall physical activity (min/day)	71	269.9	(74.6)
**Physical fitness**			
*V*O_2max_ (ml/min)	71	2339.2	(657.2)
*V*O_2max_ (ml/kg/min)	71	30.5	(5.6)
Extension peak torque (Nm)	71	265.2	(84.8)
Extension peak torque/weight (Nm/kg)	71	3.5	(0.7)
Flexion peak torque (Nm)	71	123.1	(44.4)
Flexion peak torque/weight (Nm/kg)	71	1.6	(0.5)
Hand grip strength (kg)	73	71.0	(23.7)
Hand grip strength/weight	73	0.9	(0.2)

*SD* standard deviation, *PSQI* Pittsburgh Sleep Quality Index.

**Figure 1:**
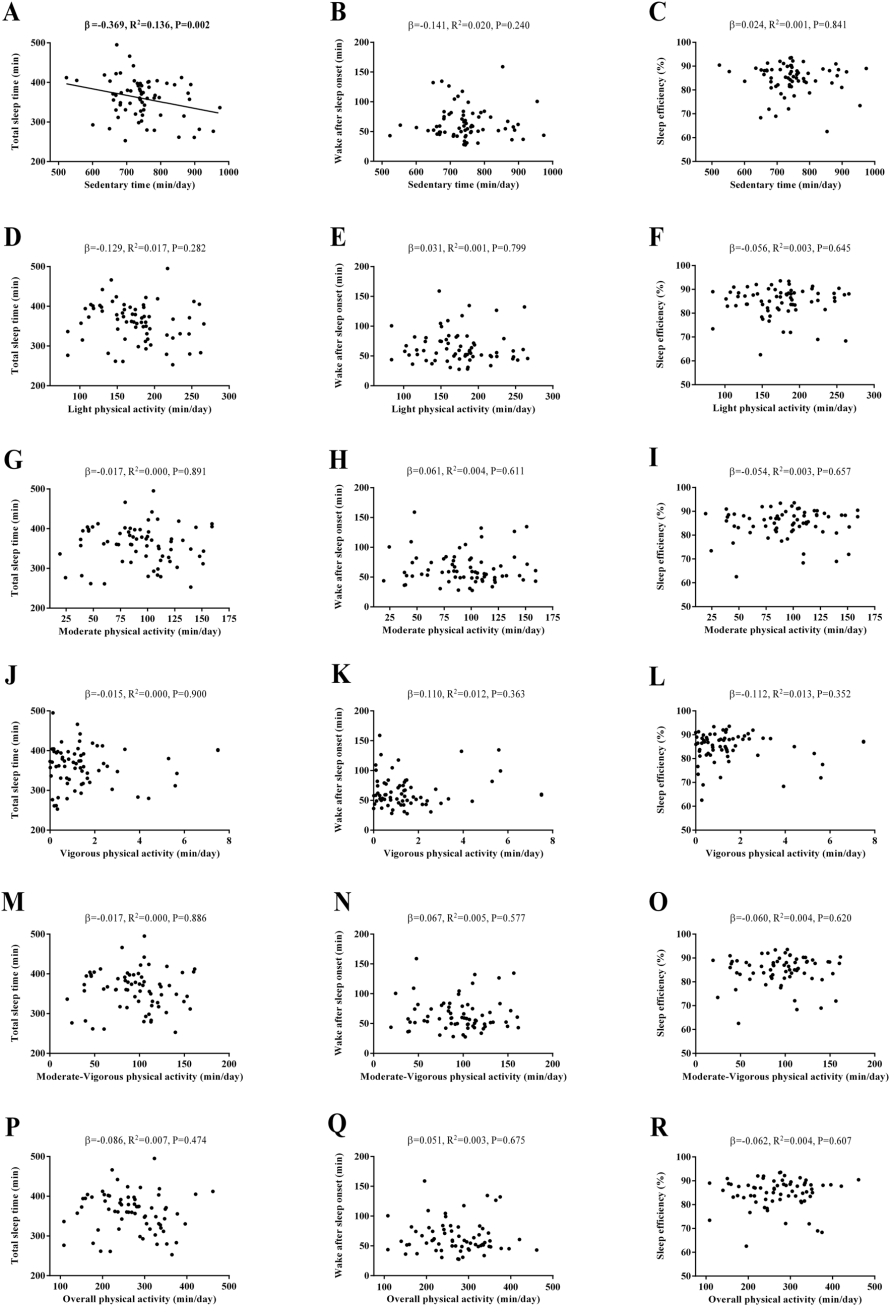
Association of physical activity levels with objective sleep quantity and quality in sedentary middle-aged adults. *β* (standardized regression coefficient), *R*^*2*^, and *P* from a simple linear regression analysis. Significant *P* values (≤ 0.05) are in bold.

Figure 1 shows the association between sedentariness and physical activity levels with objective sleep quantity and quality. A negative association was observed between sedentary time and TST (*β* = − 0.369, *R*^*2*^ = 0.136, *P* = 0.002, Fig. 1A). We did not observe any significant association between sedentary time and WASO or SE (all *P* > 0.05, Fig. 1B,C). No associations between LPA, MPA, VPA, MVPA, and overall physical activity with TST, WASO, and SE were found (all *P* > 0.05, Fig. 1D–R).

**Figure 2:**
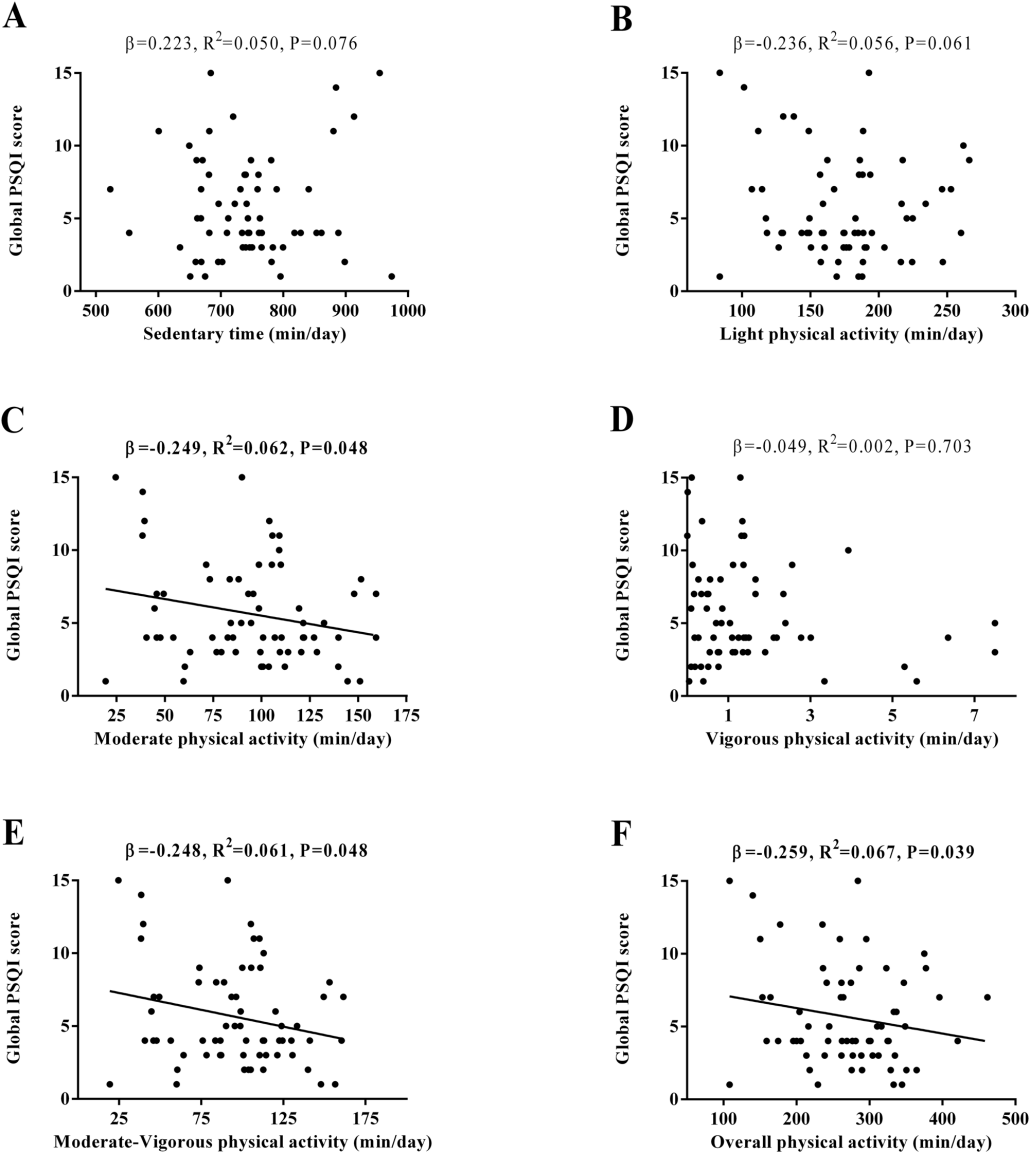
Association of physical activity levels with subjective sleep quantity and quality in sedentary middle-aged adults. *β* (standardized regression coefficient), *R*^*2*^, and *P* from a simple linear regression analysis. Significant *P* values (≤ 0.05) are in bold. *PSQI* Pittsburgh Sleep Quality Index.

Figure 2 shows the association between sedentary and physical activity levels with subjective sleep quantity and quality. We did not observe any significant association between sedentary time, LPA, and VPA with global PSQI score (all *P* > 0.05, Fig. 2A, B,D). MPA, MVPA, and overall physical activity levels were negatively associated with global PSQI score (*β* = − 0.249, *R*^*2*^ = 0.062, *P* = 0.048, Fig. 2C; *β* = − 0.248, *R*^*2*^ = 0.061, *P* = 0.048, Fig. 2E; *β* = − 0.259, *R*^*2*^ = 0.067, *P* = 0.039, Fig. 2F, respectively).

**Figure 3:**
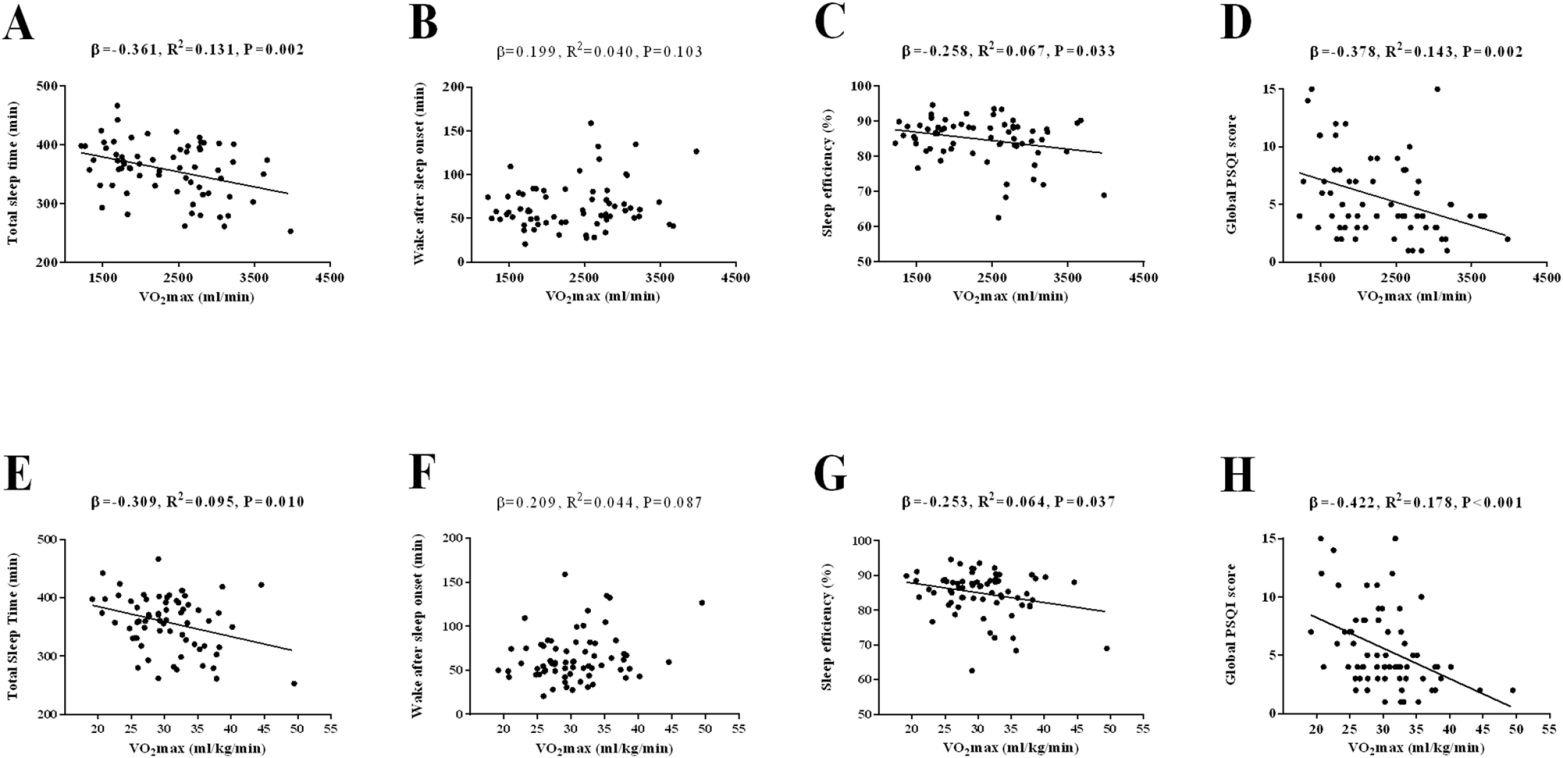
Association of cardiorespiratory fitness with both objective and subjective sleep quantity and quality in sedentary middle-aged adults. *β* (standardized regression coefficient), *R*^*2*^, and *P* from a simple linear regression analysis. Significant *P* values (≤ 0.05) are in bold. *PSQI* Pittsburgh Sleep Quality Index.

Figure 3 shows the association between *V*O_2max_ with sleep quantity and quality. Negative associations were found between *V*O_2max_ and both TST and SE (*β* = − 0.361, *R*^*2*^ = 0.131, *P* = 0.002, Fig. 3A; *β* = − 0.258, *R*^*2*^ = 0.067, *P* = 0.033, Fig. 3C, respectively). Negative associations were found between *V*O_2max_ and both TST and SE when *V*O_2max_ was expressed relative to body weight (*β* = − 0.309, *R*^*2*^ = 0.095, *P* = 0.010, Fig. 3E; *β* = − 0.253, *R*^*2*^ = 0.064, *P* = 0.037, Fig. 3G, respectively). No association was observed between *V*O_2max_ and WASO (*P* > 0.05, Fig. 3B). No association was observed between *V*O_2max_ and WASO when *V*O_2max_ was expressed relative to body weight (*P* > 0.05, Fig. 3F). A negative association was observed between *V*O_2max_ and global PSQI score (*β* = − 0.378, *R*^*2*^ = 0.143, *P* = 0.002, Fig. 3D). A negative association was observed between *V*O_2max_ (expressed relative to body weight) and global PSQI score (*β* = − 0.422, *R*^*2*^ = 0.178, *P* < 0.001, Fig. 3H).

**Figure 4:**
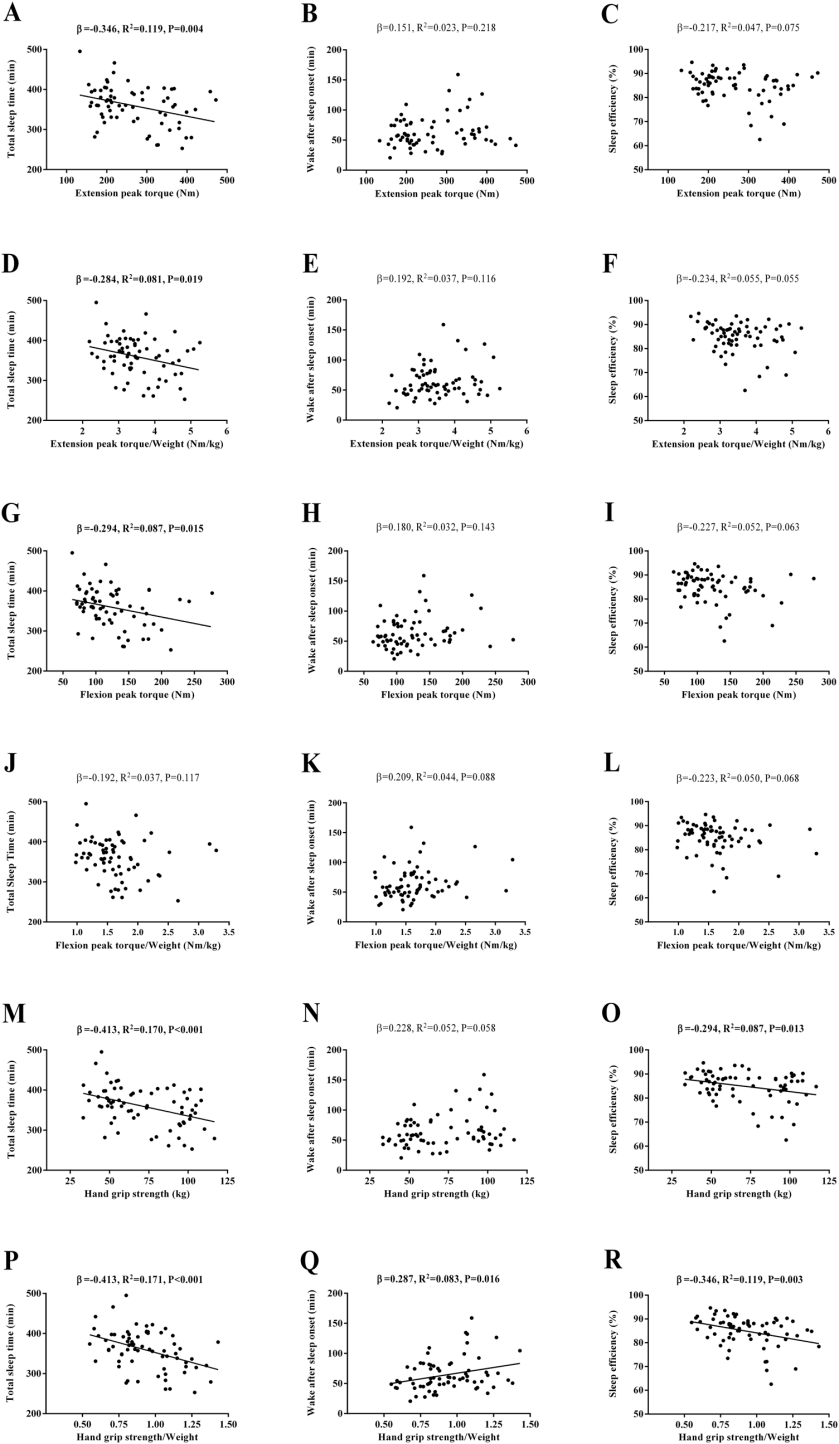
Association of muscular strength with objective sleep quantity and quality in sedentary middle-aged adults. *β* (standardized regression coefficient), *R*^*2*^, and *P* from a simple linear regression analysis. Significant *P* values (≤ 0.05) are in bold.

Figure 4 shows the association between muscular strength and objective sleep quantity and quality. Negative associations of extension peak torque, flexion peak torque, and hand grip strength with TST were observed (*β* = − 0.346, *R*^*2*^ = 0.119, *P* = 0.004, Fig. 4A; *β* = − 0.294, *R*^*2*^ = 0.087, *P* = 0.015, Fig. 4G; *β* = − 0.413, *R*^*2*^ = 0.170, *P* < 0.001, Fig. 4M, respectively). Negative associations of extension peak torque and hand grip strength with TST were observed when expressed relative to body weight (*β* = − 0.284, *R*^*2*^ = 0.081, *P* = 0.019, Fig. 4D; *β* = − 0.413, *R*^*2*^ = 0.171, *P* < 0.001, Fig. 4P, respectively). No association of flexion peak torque with TST was observed when expressed relative to body weight (P > 0.05, Fig. 4J). No associations of extension peak torque and flexion peak torque with WASO and SE were observed (all P > 0.05, Fig. 4B,C,H,I). No associations of extension peak torque and flexion peak torque with WASO and SE were observed when expressed relative to body weight (all P > 0.05, Fig. 4E,F,K,L). A negative association of hand grip strength with SE was observed (*β* = − 0.294, *R*^*2*^ = 0.087, *P* = 0.013, Fig. 4O). A negative association of hand grip strength with SE was observed when expressed relative to body weight (*β* = − 0.346, *R*^*2*^ = 0.119, *P* = 0.003, Fig. 4R). No association of hand grip strength with WASO was observed (P > 0.05, Fig. 4N). A positive association of hand grip strength with WASO was observed when expressed relative to body weight (*β* = 0.287, *R*^*2*^ = 0.083, *P* = 0.016, Fig. 4Q).

**Figure 5:**
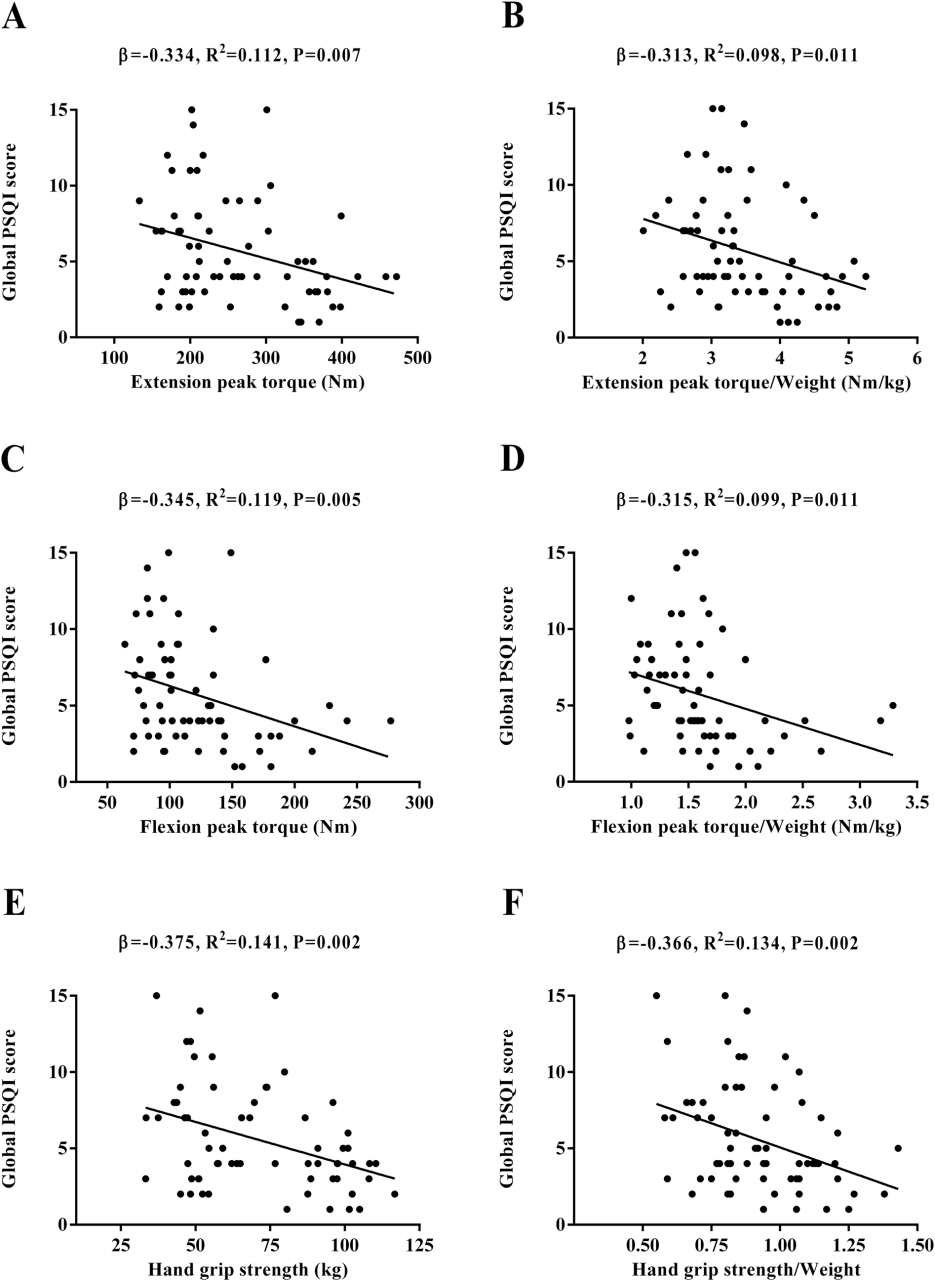
Association of muscular strength with subjective sleep quantity and quality in sedentary middle-aged adults. *β* (standardized regression coefficient), *R*^*2*^, and *P* from a simple linear regression analysis. Significant *P* values (≤ 0.05) are in bold. *PSQI* Pittsburgh Sleep Quality Index.

Figure 5 shows the association between muscular strength and subjective sleep quantity and quality. Extension peak torque, flexion peak torque, and hand grip strength were negatively associated with global PSQI score (*β* = − 0.334, *R*^*2*^ = 0.112, *P* = 0.007, Fig. 5A; *β* = − 0.345, *R*^*2*^ = 0.119, *P* = 0.005, Fig. 5C; *β* = − 0.375, *R*^*2*^ = 0.141, *P* = 0.002, Fig. 5E, respectively). Extension peak torque, flexion peak torque, and hand grip strength were negatively associated with global PSQI score when expressed relative to body weight (*β* = − 0.313, *R*^*2*^ = 0.098, *P* = 0.011, Fig. 5B; *β* = − 0.315, *R*^*2*^ = 0.099, *P* = 0.011, Fig. 5D; *β* = − 0.366, *R*^*2*^ = 0.134, *P* = 0.002, Fig. 5F, respectively).

Almost all of the above-mentioned findings persisted once age, fat mass percentage, FMI, LMI and alcohol intake were included in the statistical models (Supplementary material Tables S1 and S2).

## Discussion

Our study was aimed at investigating the association of sedentariness, physical activity levels and fitness with sleep quantity and quality in sedentary middle-aged adults. Results from our study showed that reduced levels of sedentariness, greater *V*O_2max_, and greater muscular strength were positively related to improved objective sleep quantity and quality. Furthermore, higher levels of overall physical activity, *V*O_2max_, and muscular strength were related to better subjective sleep quantity and quality.

Physical activity has been postulated as an effective tool to improve sleep quantity and quality^27^, mainly due to its regulatory role on the circadian rhythmicity^39^. Indeed, poor levels of physical activity and a pattern of sedentary behaviour have both been proposed as important risk factors for insomnia and sleep disturbances in adults^30^.

A systematic review and meta-analysis by Kredlow et al.^27^—which examined the effects of acute and regular exercise on a range of sleep outcomes including 66 studies in the analyses and 2863 participants of all ages—found small to medium significant beneficial effects of acute and regular exercise on TST, SE, and WASO, and a large positive impact of regular exercise on overall subjective sleep quality. Interestingly, it was reported that the impact of exercise on objective sleep outcomes was higher in young adults. This fact could explain the observed non-significant associations between physical activity and objective sleep outcomes in our study sample (i.e. older adults). In this sense, a recently published original study by Mitchell et al.^40^ including 353 women also reported no evidence of a significant relationship between physical activity and sleep quantity and quality both measured by accelerometry. Moreover, Sloan et al.^41^ examined the independent, joint, and fully combined associations of objectively measured sedentary behaviour and MVPA with self-reported sleep quality in 757 healthy working adults aged 21–64 years old. They showed that sedentary behaviour and MVPA levels were not significantly associated with sleep quality, which concur with the present findings.

Regarding intensity of physical activity (i.e. LPA, MPA, VPA, and MVPA), and sedentary time, we found statistically significant associations of each physical activity intensity and sedentariness with TST, WASO and SE after adjusting by fat mass percentage, FMI and/or LMI, these variables therefore being potential moderators of the impact of physical activity on sleep. These results are essential as the role of intensity of physical activity on the relationship between physical activity and sleep both measured by an objective method (i.e. accelerometry) had not yet been robustly studied and therefore clarified.

Gathered research has well-established that *V*O_2max_ is a powerful marker of health and longevity^10^. As the prevalence of sleep disturbances significantly increases in older adults^42^, maintaining a correct level of *V*O_2max_ while aging may also have beneficial effects on sleep quality, reducing therefore the incidence of sleep disorders in the elderly. Dishman et al.^43^ suggested that the maintenance of an appropriated cardiorespiratory fitness during middle-age—when the decline in fitness typically accelerates and an increased risk of developing sleep disturbances appears—may be a protective factor against the onset of sleep complaints.

However, there is a reduced number of available studies measuring *V*O_2max_ and sleep quantity and quality parameters using reliable and/or gold-standard methods and specifically including healthy samples, who have not yet suffered the development of degenerative diseases caused by the aging process. Thus, studying the association between *V*O_2max_ and sleep quantity and quality in healthy samples using reliable measurements could play an important role in the prevention of the most common sleep disorders, i.e. insomnia and obstructive sleep apnoea.

In our study, we found that higher levels of *V*O_2max_ were associated with both improved objective sleep quantity and quality (greater TST and SE) and better subjective sleep quantity and quality. Our results support those found in previous studies where lower *V*O_2max_ was associated to worse sleep quality and insomnia. Strand et al.^32^ showed an inverse association between subjectively measured insomnia and VO_2_peak, independently to self-reported physical activity. Similarly, Zou et al.^33^ also reported that insomnia was related to lower *V*O_2max_ in middle-aged men (aged 50–64 years), independently to body composition, living conditions, comorbidities, and lifestyles.

Muscular strength is considered to be a powerful predictor of health and all-cause mortality^9^. According to previous research^44^, lower levels of muscular strength may be an important risk factor for poor sleep quality in middle-aged adults, related to severe sleep disorders such as obstructive sleep apnoea, which is characterised by repetitive events of upper airway collapse during sleep due to atony of respiratory muscles.

To the best of our knowledge, the association between muscular strength and sleep quantity and quality has not been accurately studied yet in healthy sedentary middle-aged adults using gold-standard methods such as accelerometry. Furthermore, results found in this field of research are still inconsistent due to several reasons such as different study designs, confounder variables (e.g. age and sex), and different measurements of sleep duration.

Furthermore, our results showed that higher levels of muscular strength were associated with better objective and subjective sleep quantity and quality in sedentary middle-aged adults. These results support the findings obtained by previous studies^45^ where hand grip strength was associated with subjective sleep quality. Our results also extend those from other previous studies where sleep was only subjectively measured. Chen et al.^34^ found that in older adults (aged 65 years and older) hand grip strength differed between sleep duration groups, observing that short and long sleepers had weaker hand grip strength than the mid-range sleepers. Similarly, Wang et al.^35^ studied a middle-aged and older population obtaining that shorter or longer sleep may predict a weaker follow-up grip strength and a faster rate of hand grip strength decline over time compared to intermediate sleep duration. The analysis of these potential differences in muscular strength depending on short, intermediate and long sleep duration was not possible in our study due to the lack of the long sleepers group in our sample.

Our study has significant implications for research and clinical practice. Consistent with past research, we demonstrated that physical activity, cardiorespiratory fitness and muscular strength, although unrelated to sleep quantity, have a beneficial effect on sleep quality in healthy sedentary middle-aged adults, as well as sedentariness having a negative impact on sleep-related parameters. Thus, increased physical activity and physical fitness may be a potential strategy to prevent and/or treat sleep disturbances. Our inclusion of the gold-standard measure of sedentariness and physical activity, however, as well as an objective measure of sleep (i.e. accelerometry), provide a more reliable and robust perspective on this field of research.

Our results regarding the impact of physical activity on perceived sleep quality are noteworthy as subjective sleep quality has been related to a vast number of outcomes such as well-being and successful aging, cognitive decline, daytime functioning, and mental health in healthy individuals. Furthermore, perceived poor sleep quality in patients with insomnia has been shown to contribute to the maintenance of sleep disturbances^46^ and, therefore, may be an important target in interdisciplinary interventions.

Our findings, however, must be interpreted with caution as they are limited to the sample included and study design. Firstly, the cross-sectional study design does not allow to establish causal inferences, so future well-designed longitudinal studies are needed in order to clarify causality. Regarding the sample, our study only included healthy sedentary middle-aged adults, so generalization of results to a wider population may not be possible. Although accelerometry was used as an objective measure of sleep quantity and quality—which has been shown to be as reliable as the gold standard measure for sleep, i.e. polysomnography, when associating physical activity and sleep—it may overestimate TST and SE, as well as underestimate sleep onset latency and WASO in adults^31^. Thus, future research should include, apart from accelerometry as an objective measure of physical activity, polysomnography as the most reliable measure of sleep quantity and quality, also providing data on more specific sleep outcomes such as sleep architecture (i.e. rapid eye movement [REM] sleep stage, and nonREM sleep stages [N1, N2, and N3]).

## Supplementary Information

Supplementary Information
