# Supplementary material for: Role of physical activity and fitness on sleep in sedentary middle-aged adults: the FIT-AGEING study
Source: Sci Rep. 2021 Jan 12;11:539. doi: 10.1038/s41598-020-79355-2 (PMC7804461; doi:10.1038/s41598-020-79355-2)
Supplement: Supplementary file 1 — Supplementary Information [file 41598_2020_79355_MOESM1_ESM.pdf]

Role of physical activity and fitness on sleep in sedentary middle-aged adults: The FIT-AGEING study

Sol Mochón-Benguigui[1*†], Almudena Carneiro-Barrera[2†], Manuel J. Castillo[1], Francisco J. Amaro-Gahete[1*]

[1] EFFECTS-262 Research group, Department of Medical Physiology, School of Medicine, University of Granada, Granada 18016, Spain

[2] Sleep and Health Promotion Laboratory, Mind, Brain and Behaviour Research Centre (CIMCYC), University of Granada, Granada 18011, Spain

*Corresponding author: Francisco J. Amaro-Gahete (amarof@ugr.es)
& Sol Mochón-Benguigui (solmb@correo.ugr.es)

[†]These two authors equally contributed to this work.

SUPPLEMENTARY MATERIAL

Table S1. Association of physical activity levels with sleep quantity and quality (Model 0) adjusted by age (Model 1), by total fat mass percentage (Model 2), by fat mass index (Model 3) and by lean mass index (Model 4).

	**Total sleep time**			**Wake after sleep onset**			**Sleep efficiency**			**Global PSQI score**		
	ß	R^2	P	ß	R^2	P	ß	R^2	P	ß	R^2	P
Sedentary time (min/day)												
Model 0	-0.369	0.136	**0.002**	-0.141	0.020	0.240	0.024	0.001	0.841	0.223	0.050	0.076
Model 1	-0.368	0.142	**0.006**	-0.141	0.020	0.496	0.024	0.001	0.964	0.228	0.095	**0.048**
Model 2	-0.327	0.280	**<0.001**	-0.178	0.134	**0.007**	0.066	0.145	**0.005**	0.251	0.168	**0.004**
Model 3	-0.351	0.206	**<0.001**	-0.160	0.094	**0.035**	0.044	0.090	**0.041**	0.231	0.073	0.100
Model 4	-0.329	0.257	**<0.001**	-0.171	0.085	**0.049**	0.059	0.089	**0.042**	0.268	0.264	**<0.001**
Light physical activity (min/day)												
Model 0	-0.129	0.017	0.282	0.031	0.001	0.799	-0.056	0.003	0.645	-0.236	0.056	0.061
Model 1	-0.134	0.024	0.431	0.030	0.001	0.952	-0.055	0.003	0.888	-0.234	0.098	**0.043**
Model 2	-0.141	0.194	**0.001**	0.039	0.104	**0.024**	-0.066	0.145	**0.005**	-0.229	0.158	**0.005**
Model 3	-0.139	0.102	**0.025**	0.039	0.070	0.085	-0.065	0.092	**0.037**	-0.232	0.073	0.098
Model 4	-0.135	0.168	**0.002**	0.034	0.057	0.135	-0.060	0.089	**0.042**	-0.239	0.250	**<0.001**
Moderate physical activity (min/day)												
Model 0	-0.017	0.000	0.891	0.061	0.004	0.611	-0.054	0.003	0.657	-0.249	0.062	**0.048**
Model 1	-0.006	0.007	0.800	0.066	0.005	0.848	-0.058	0.004	0.878	-0.223	0.092	0.053
Model 2	0.027	0.175	**0.001**	0.028	0.103	**0.024**	-0.015	0.141	**0.006**	-0.206	0.147	**0.008**
Model 3	0.006	0.083	0.052	0.041	0.070	0.084	-0.031	0.089	**0.042**	-0.236	0.075	0.094
Model 4	0.021	0.150	**0.004**	0.039	0.058	0.133	-0.026	0.086	**0.046**	-0.197	0.231	**<0.001**
Vigorous physical activity (min/day)												
Model 0	-0.015	0.000	0.900	0.110	0.012	0.363	-0.112	0.013	0.352	-0.049	0.002	0.703
Model 1	-0.004	0.007	0.801	0.115	0.014	0.629	-0.117	0.014	0.618	-0.021	0.044	0.257
Model 2	0.006	0.174	**0.001**	0.094	0.111	**0.018**	-0.093	0.149	**0.004**	-0.027	0.106	**0.033**
Model 3	-0.039	0.085	**0.050**	0.131	0.086	**0.048**	-0.137	0.107	**0.022**	-0.055	0.023	0.498
Model 4	0.078	0.156	**0.003**	0.058	0.059	0.125	-0.047	0.088	**0.044**	0.037	0.194	**0.001**
Moderate-vigorous physical activity (min/day)												
Model 0	-0.017	0.000	0.886	0.067	0.005	0.577	-0.060	0.004	0.620	-0.248	0.061	**0.048**
Model 1	-0.006	0.007	0.800	0.072	0.006	0.823	-0.064	0.005	0.855	-0.221	0.091	0.054
Model 2	0.027	0.175	**0.001**	0.034	0.104	**0.024**	-0.021	0.141	**0.006**	-0.205	0.146	**0.008**
Model 3	0.003	0.083	0.052	0.049	0.071	0.082	-0.039	0.089	**0.041**	-0.235	0.074	0.095
Model 4	0.025	0.151	**0.004**	0.042	0.058	0.132	-0.028	0.086	**0.046**	-0.192	0.229	**<0.001**
Total physical activity (min/day)												
Model 0	-0.086	0.007	0.474	0.051	0.003	0.675	-0.062	0.004	0.607	-0.259	0.067	**0.039**
Model 1	-0.084	0.014	0.630	0.051	0.003	0.896	-0.063	0.004	0.859	-0.244	0.102	**0.037**
Model 2	-0.072	0.180	**0.001**	0.040	0.104	**0.024**	-0.049	0.143	**0.005**	-0.234	0.159	**0.005**
Model 3	-0.082	0.090	**0.041**	0.047	0.071	0.083	-0.058	0.091	**0.039**	-0.250	0.082	0.074
Model 4	-0.070	0.155	**0.003**	0.041	0.058	0.133	-0.050	0.088	**0.043**	-0.233	0.247	**<0.001**

β (standardized regression coefficient), R^2, and *P*-value of simple and multiple-regression analysis.

Significant P values (< 0.05) are in bold. PSQI: Pittsburgh Sleep Quality Index.

Table S2. Association of physical fitness (including cardiorespiratory fitness and muscular strength) with sleep quantity and quality (Model 0) adjusted by age (Model 1), by total fat mass percentage (Model 2), by fat mass index (Model 3) and by lean mass index (Model 4).

	**Total sleep time**			**Wake after sleep onset**			**Sleep efficiency**			**Global PSQI score**		
	ß	R^2	P	ß	R^2	P	ß	R^2	P	ß	R^2	P
VO_2max (ml/min)												
Model 0	-0.361	0.131	**0.002**	0.199	0.040	0.103	-0.258	0.067	**0.033**	-0.378	0.143	**0.002**
Model 1	-0.365	0.132	**0.010**	0.202	0.040	0.261	-0.263	0.069	0.097	-0.362	0.163	**0.004**
Model 2	-0.234	0.173	**0.002**	0.072	0.082	0.061	-0.119	0.118	**0.017**	-0.291	0.161	**0.004**
Model 3	-0.335	0.171	**0.002**	0.172	0.081	0.063	-0.228	0.118	**0.017**	-0.366	0.148	**0.007**
Model 4	-0.252	0.136	**0.009**	0.090	0.046	0.220	-0.148	0.073	0.086	-0.082	0.187	**0.002**
VO_2max (ml/kg/min)												
Model 0	-0.309	0.095	**0.010**	0.209	0.044	0.087	-0.253	0.064	**0.037**	-0.422	0.178	**<0.001**
Model 1	-0.314	0.097	**0.036**	0.213	0.045	0.226	-0.260	0.067	0.105	-0.404	0.193	**0.001**
Model 2	-0.129	0.143	**0.007**	0.052	0.080	0.066	-0.075	0.111	**0.022**	-0.362	0.184	**0.002**
Model 3	-0.247	0.107	**0.026**	0.125	0.064	0.117	-0.165	0.087	0.052	-0.458	0.183	**0.002**
Model 4	-0.193	0.145	**0.006**	0.144	0.059	0.137	-0.172	0.089	**0.049**	-0.275	0.241	**<0.001**
Extension peak torque (Nm)												
Model 0	-0.346	0.119	**0.004**	0.151	0.023	0.218	-0.217	0.047	0.075	-0.334	0.112	**0.007**
Model 1	-0.346	0.119	**0.016**	0.167	0.031	0.359	-0.233	0.056	0.155	-0.304	0.131	**0.013**
Model 2	-0.183	0.180	**0.002**	-0.003	0.077	0.073	-0.044	0.115	**0.019**	-0.233	0.127	**0.015**
Model 3	-0.305	0.160	**0.003**	0.108	0.070	0.093	-0.169	0.104	**0.028**	-0.324	0.112	**0.025**
Model 4	-0.123	0.143	**0.007**	0.033	0.030	0.375	-0.061	0.059	0.139	0.074	0.180	**0.002**
Extension peak torque/Weight (Nm/kg)												
Model 0	-0.284	0.081	**0.019**	0.192	0.037	0.116	-0.234	0.055	0.055	-0.313	0.098	**0.011**
Model 1	-0.284	0.081	**0.064**	0.210	0.047	0.211	-0.252	0.064	0.115	-0.283	0.119	**0.020**
Model 2	-0.029	0.157	**0.004**	0.006	0.077	0.073	-0.009	0.114	**0.019**	-0.191	0.112	**0.025**
Model 3	-0.199	0.098	**0.035**	0.085	0.064	0.115	-0.118	0.087	0.053	-0.368	0.103	**0.034**
Model 4	-0.117	0.148	**0.006**	0.141	0.043	0.237	-0.147	0.073	0.085	-0.105	0.186	**0.002**
Flexion peak torque (Nm)												
Model 0	-0.294	0.087	**0.015**	0.180	0.032	0.143	-0.227	0.052	0.063	-0.345	0.119	**0.005**
Model 1	-0.292	0.088	0.051	0.186	0.038	0.282	-0.233	0.057	0.149	-0.325	0.147	**0.007**
Model 2	-0.103	0.164	**0.003**	0.032	0.078	0.071	-0.052	0.116	**0.018**	-0.250	0.133	**0.012**
Model 3	-0.237	0.122	**0.015**	0.118	0.072	0.088	-0.159	0.100	**0.033**	-0.339	0.119	**0.020**
Model 4	-0.063	0.140	**0.007**	0.117	0.036	0.302	-0.114	0.064	0.115	-0.073	0.181	**0.002**
Flexion peak torque/Weight (Nm/kg)												
Model 0	-0.192	0.037	0.117	0.209	0.044	0.088	-0.223	0.050	0.068	-0.315	0.099	**0.011**
Model 1	-0.190	0.039	0.275	0.212	0.049	0.197	-0.226	0.054	0.166	-0.302	0.133	**0.012**
Model 2	0.070	0.160	**0.003**	0.066	0.080	0.066	-0.032	0.115	**0.019**	-0.202	0.119	**0.019**
Model 3	-0.067	0.073	0.084	0.108	0.067	0.104	-0.101	0.084	0.058	-0.348	0.101	**0.036**
Model 4	-0.063	0.142	**0.007**	0.168	0.054	0.166	-0.155	0.079	0.070	-0.159	0.199	**0.001**
Hand grip strength (kg)												
Model 0	-0.413	0.170	**<0.001**	0.228	0.052	0.058	-0.294	0.087	**0.013**	-0.375	0.141	**0.002**
Model 1	-0.408	0.171	**0.002**	0.233	0.054	0.158	-0.301	0.089	**0.044**	-0.350	0.165	**0.003**
Model 2	-0.256	0.212	**<0.001**	0.068	0.096	**0.034**	-0.120	0.139	**0.007**	-0.292	0.151	**0.005**
Model 3	-0.368	0.210	**<0.001**	0.181	0.096	**0.034**	-0.243	0.140	**0.006**	-0.365	0.142	**0.007**
Model 4	-0.341	0.172	**0.002**	0.160	0.053	0.160	-0.233	0.088	**0.046**	0.087	0.195	**0.001**
Hand grip strength/Weight												
Model 0	-0.413	0.171	**<0.001**	0.287	0.083	**0.016**	-0.346	0.119	**0.003**	-0.366	0.134	**0.002**
Model 1	-0.409	0.173	**0.002**	0.291	0.084	0.053	-0.349	0.121	**0.013**	-0.345	0.163	**0.003**
Model 2	-0.233	0.193	**0.001**	0.129	0.100	**0.030**	-0.167	0.141	**0.006**	-0.300	0.137	**0.009**
Model 3	-0.370	0.175	**0.002**	0.211	0.095	**0.035**	-0.267	0.133	**0.008**	-0.425	0.142	**0.008**
Model 4	-0.285	0.191	**0.001**	0.257	0.084	0.054	-0.293	0.123	**0.012**	-0.107	0.199	**0.001**

β (standardized regression coefficient), R^2, and *P*-value of simple and multiple-regression analysis. Significant P values (< 0.05) are in bold. PSQI: Pittsburgh Sleep Quality Index.
